# A Brazilian Portuguese cross-cultural adaptation of the modified JOA scale for myelopathy

**DOI:** 10.6061/clinics/2017(02)06

**Published:** 2017-02

**Authors:** Raphael R. Pratali, Justin S. Smith, Rodrigo L.N. Motta, Samuel M. Martins, Marcel M. Motta, Ricardo D. Rocha, Carlos Fernando P.S. Herrero

**Affiliations:** I Hospital do Servidor Público Estadual de São Paulo, Ortopedia e Traumatologia, São Paulo/SP, Brazil; II University of Virginia Health System, Department of Neurosurgery Charlottesville, VA, United States; III Universidade de São Paulo, Faculdade de Medicina de Ribeirão Preto, Departamento de Biomecânica, Medicina e Reabilitação do Aparelho Locomotor, Universidade de São Paulo, Ribeirão Preto/SP, Brazil

**Keywords:** Cervical Myelopathy, Outcome Assessment, Scales, Translating

## Abstract

**OBJECTIVES:**

To develop a version of the modified Japanese Orthopaedic Association (mJOA) scale that had been translated into Portuguese and cross-culturally adapted for the Brazilian population.

**METHODS:**

The well-established process of forward-backward translation was employed along with cross-cultural adaptation.

**RESULTS:**

Three bilingual translators (English and native Portuguese) performed the forward translation of the mJOA scale from English to Portuguese based on iterative discussions used to reach a consensus translation. The translated version of the mJOA scale was then back-translated into English by a native English-speaking translator unaware of the concepts involved with the mJOA scale. The original mJOA scale and the back-translated version were compared by a native North American neurosurgeon, and as they were considered equivalent, the final version of the mJOA scale that had been translated into Portuguese and cross-culturally adapted was defined.

**CONCLUSION:**

To facilitate global and cross-cultural comparisons of the severity of cervical myelopathy, this study presents a version of the mJOA scale that was translated into Portuguese and cross-culturally adapted for the Brazilian population.

## INTRODUCTION

Although cervical myelopathy may result from a variety of congenital and acquired pathologies, it most commonly results from spondylotic spinal cord compression 1-3. Cervical myelopathy can be associated with neurological deficits, which may result in different degrees of disability, varying from mild to severe 4. Therapeutic decision-making for cervical myelopathy is also variable, with the primary aim of treatment being the prevention of disease progression.

Due to such variation in the degree of neurological involvement and severity of disability, mechanisms have been developed to objectively characterize the clinical presentation and to grade disease severity. In 1985, a scale for cervical myelopathy severity was developed by the Japanese Orthopedic Association (JOA). The JOA scale uses a scoring system that separately addresses the function of the upper and lower limbs, sensory deficits and sphincter dysfunction 5. However, the original version evaluated the degree of involvement of the upper limbs based on the ability to use “chopsticks”, which has limited its application in Western populations. Therefore, the JOA scale was modified (mJOA) to assess the degree of involvement of the upper limbs with other manual abilities 6.

The universal use of the same scale allows comparisons of the same pathology across different populations and facilitates an analysis of the effect of the pathology in various locations and the results after different treatment approaches. The mJOA scale, however, was originally published in English, and limitations exist regarding the use of this scale in individuals who do not comprehend the English language and in populations with cultural differences 7. Thus, the questionnaires and scales must be not only translated but also culturally adapted to populations 8. The mJOA scale was translated and validated for the Dutch population in 2010 to encourage its adoption in other populations and cultures in the Western hemisphere 9, but to date, no Portuguese version is available that has been culturally adapted for the Brazilian population. The purpose of the present study was to develop a version of the mJOA scale that has been translated into Portuguese and cross-culturally adapted for the Brazilian population.

## MATERIALS AND METHODS

The present study presents the translation and cross-cultural adaptation of the mJOA questionnaire through the collaborative efforts of two centers that specialize in the treatment of spinal disorders. The well-established process of forward-backward translation was employed 10,11. This methodical approach helped ensure the quality of the translation and adaptation and has been utilized for several questionnaires used to assess spinal pathology 11.

### Initial Translation into the Brazilian Portuguese Language

The forward translation of the originally presented mJOA scale (Table 1) was performed by three bilingual translators (Brazilian Portuguese and English) whose native language was Brazilian Portuguese. Two of these translators were spine surgeons (RRP and CFPSH), and the third was a physiotherapist (RLNM). All translators were aware of the study objectives. These three translators initially worked independently, and the translated versions were then compared and discussed until a consensus synthesized translation was obtained.

### Back Translation

The synthesized translated version of the mJOA scale was then back-translated into English by a fourth bilingual translator (Brazilian Portuguese and English) whose native language was English and who did not participate in the initial translation. The translator who provided the back translation to English was blinded to the original English version of the mJOA scale and was unaware of the concepts involved with the mJOA scale. The purpose of this back translation was to identify any inconsistencies or conceptual errors in translation 10.

### Reviewer Committee

The original mJOA scale and the versions obtained by the forward and back translation were analyzed by a multidisciplinary committee consisting of four bilingual specialists (the three translators who provided the initial translation into Portuguese and the translator who provided back translation into English). All reviewers were informed about the measurements and concepts involved with the mJOA scale. Each committee member evaluated the texts for equivalency and any discrepancies between the two versions in four specific areas: semantic, idiomatic, experiential and conceptual.

## RESULTS

The three independent versions resulting from initial forward translation by each of the translators had limited semantic and idiomatic discrepancies, but no evidence of conceptual discrepancies was found. These discrepancies were compared and discussed in a videoconference meeting among the translators, and a consensus was reached for a translated version. The back translation resulted in a version with limited semantic and idiomatic discrepancies with the original mJOA scale, but the back-translated version was conceptually similar to the original mJOA scale. These discrepancies were discussed in the reviewer committee, and the version translated into Portuguese was adapted to address these discrepancies. The final back-translated version of the translated and adapted Portuguese version of the mJOA scale was analyzed and compared with the original mJOA scale by a native North American, English-speaking neurosurgeon (JSS), and as the versions were considered equivalent. This version that had been translated to Portuguese and cross-culturally adapted for the Portuguese-speaking Brazilian population was considered the definitive version of the mJOA scale (Table 2).

## DISCUSSION

The vast majority of scales and questionnaires designed to evaluate the effect of cervical conditions on function and quality of life have been developed in languages other than Portuguese. The importance of adapting standardized clinical assessment questionnaires into different languages to encourage their broader application 9 and to help standardize and facilitate the exchange of information within the scientific community has been well-recognized 11,12.

The JOA proposed a scale to objectively assess the severity of cervical myelopathy in both the clinical and post-operative treatment settings for the Japanese population 5. Intending to apply this particular assessment tool to a Western population, Benzel et al. developed an mJOA scale that was translated into English and cross-culturally adapted, including changing references from chopsticks to spoons 6. The cross-cultural adaptation of a translated questionnaire or scale is an important step in this process because some expressions may be commonly used in the original language and culture but may not be similarly used or understood in the country or language in which the translated version will be applied.

To help stimulate the global adoption of spine outcome tools originally developed in English, the mJOA scale was translated and adapted for the Dutch population 9. To date, the present study is the first to translate the mJOA scale into the Portuguese language and to provide cross-cultural adaptation of this translated version for the Brazilian population. No difficulties in adjusting expressions from the original scale in English were encountered in the process of translation to Brazilian Portuguese. Because the mJOA scale is administered by healthcare personnel on behalf of patients, less vulnerability exists for cross-cultural discrepancies in sentences addressing the function of the arms, legs, and micturition because these professionals are typically more familiar with these terms in their practice.

The mJOA scale, regardless of language, has intrinsic limitations. For example, in the mJOA scale, the severity of myelopathy is based on the subjective interpretation of terms such as “great difficulty” or “slight difficulty” and “severe sensory loss” or “mild sensory loss”. Bartels et al. highlighted that neither the English version nor the Dutch version of the mJOA scale has been validated as a measuring instrument 9.

The present study provides a version of the mJOA scale that was translated to Portuguese and cross-culturally adapted for the Brazilian population. This translated version can help facilitate the objective assessment of cervical myelopathy in the Portuguese-speaking Brazilian population and enable comparisons of the findings across different populations and cultures.

## AUTHOR CONTRIBUTIONS

Pratali RR conceived and designed the study, was responsible for the translation procedures, data analysis and interpretation, and manuscript drafting. Smith JS conceived and designed the study and was responsible for data analysis and interpretation, and critical revision of the manuscript. Martins SM, Motta MM and Rocha RD were responsible for data acquisition and ERB approval. Motta RL was responsible for the translation procedures. Herrero CF was responsible for the translation procedures, data analysis and interpretation and critical revision of the manuscript. All of the authors reviewed the final version of the manuscript before submission.

## Figures and Tables

**Table:** Model of the original English version of the mJOA scale that was considered in the translation process.

SCORE	DEFINITION
	Motor dysfunction score of the upper extremities
0	Inability to move hands
1	Inability to eat with a spoon, but able to move hands
2	Inability to buttom shirt, but able to eat with a spoon
3	Able to button shirt with great difficulty
4	Able to button shirt with slight difficulty
5	No dysfunction
	Motor dysfunction score of the lower extremities
0	Complete loss of motor and sensory function
1	Sensory preservation without ability to move legs
2	Able to move legs, but unable to walk
3	Able to walk on flat floor with a walking aid (i.e., cane or crutch)
4	Able to walk up and/or down stairs with hand rail
5	Moderate to significant lack of stability, but able to walk up and/or down stairs without hand rail
6	Mild lack of stability but walks with smooth reciprocation unaided
7	No dysfunction
	Sensory dysfunction score of the upper extremities
0	Complete loss of hand sensation
1	Severe sensory loss or pain
2	Mild sensory loss
3	No sensory loss
	Sphincter dysfuntion score
0	Inability to micturate voluntarily
1	Marked difficulty with micturition
2	Mild to moderate difficulty with micturition
3	Normal micturation

**Table:** Version of the mJOA scale translated to Portuguese and cross-culturally adapted for the Brazilian population.

NOTA	DEFINIÇÃO
	Escore de disfunção motora das extremidades superiores
0	Incapacidade de mover as mãos
1	Incapacidade de comer com uma colher, mas capaz de mover as mãos
2	Incapacidade de abotoar a camisa, mas capaz de comer com uma colher
3	Capaz de abotoar a camisa com grande dificuldade
4	Capaz de abotoar a camisa com pequena dificuldade
5	Sem disfunção
	Escore de disfunção motora das extremidades inferiores
0	Perda completa das funções motoras e sensitivas
1	Preservação sensitiva sem capacidade de mover as pernas
2	Capaz de mover as pernas, mas incapaz de andar
3	Capaz de andar em piso plano com auxilio para deambulação (ex. bengala ou muleta)
4	Capaz de subir e/ou descer escadas com corrimão
5	Falta de estabilidade moderada a significante, mas capaz de subir e/ou descer escadas sem corrimão
6	Falta de estabilidade leve mas anda com movimentos alternados regulares sem auxílio
7	Sem disfunção
	Escore de disfunção sensitiva das extremidades superiores
0	Perda completa da sensibilidade da mão
1	Perda sensitiva grave ou dor
2	Perda sensitiva leve
3	Sem perda sensitiva
	Escore de disfunção esfincteriana
0	Incapacidade de micção voluntaria
1	Dificuldade acentuada para micção
2	Disfunção leve a moderada para micção
3	Micção normal
